# Croupy Inflammation in the Margin of the Gums, and Its Effects

**Published:** 1862-01

**Authors:** 

**Affiliations:** Vienna


### CROUPY INFAMMATION IN THE MARGIN OF THE GUMS, AND ITS EFFECTS.

By DR. STEINBERGER, of Vienna.

Translated by George Henry, M. C. D. E.

Croupy inflammation of the gums is simply referred to by most authors, in their description of diseases of the mucous membrane of the mouth and throat, as 'diphtheritis membranacea;' and it is apparently regarded by the practical surgeon as of less importance than when it attacks the throat, because he does not see in it so much danger to the life of the individual: and yet it merits special consideration, as well for its primary appearance in the margin of the gums, as for its morbid consequences, which may involve neighboring osseous structures, as the aveolar processes, or the jaw-bone itself. This disease is of special importance to the practical surgeon, who attends particularly to diseases of children, because it often assumes an epidemic form after scarlet fever and other eruptive diseases in hospitals for children, and even in large families: it is of equal moment to the dentist, who is mostly called upon to treat it in the later stages, when it has produced loosening of the teeth, degeneration of the alveoli, or necrosis of the jaw-bone. When the disease occurs in adults, the dentist often meets with it in its earlier stage, the patient being induced to consult him, partly owing to the pain, partly to the fetid odor arising from the affection, which is a burden to his existence.

The disease presents itself under the following appearances: The gum is inflamed, somewhat swollen, and very painful, and prone to bleed on being touched. In its early stage, the margin of the gum is found to be covered with a pale-gray, structureless membranous exudation, scarcely half a line in thickness, which sloughs off in flakes. The mucous membrane beneath is deprived of its epithelium, very vascular, and bleeds readily. The formation of the exudation commences generally externally, *i. e.* on the labial and buccal portions of the gum of the lower jaw, and often first at its boundary with the lower incisors, whence it gradually progresses until the margin of the gums is completely involved, The gums of the lower and upper jaws are seldom attacked at the same time.

The exudation takes place rapidly, often in a few hours, not seldom in the course of a night; it is accompanied with a very painful sensation, described by the patient as a drawing pain: this is increased by stopping in warm places or in bed. Feverish appearances are slight, often scarcely perceptible.

As soon as the exudation is formed, it sloughs away in an ill-smelling, ichorous mass, which corrodes the mucous membrane beneath, producing an ulcerated surface. Not unfrequently, the mucous membrane of the cheeks lying on the teeth and diseased gums is covered to a great extent with this exudation, as also the border and under surface of the tongue. An inflammatory action is likewise set up in the lining membrane of the alevoli, through the ichorous discharge; it becomes strongly injected, swollen, and covered with exudation, which, after sloughing in the alvoli, particularly in those of the lower jaw, is retained, as it were, in a reservoir. The teeth become loose through the swelling of the periosteum, appearing longer to the patient; and through the continued presence of the corroding ichor, the periosteum is destroyed, and the teeth, being disconnected with the jaw, either fall out of themselves or are readily removed.

"When the periosteum is destroyed, this exerts its corrosive power on the bones, which become infiltrated with the fluid, carious in larger or smaller portions, and finally necrosed. In the almost epidemic cases of croupy gums occurring in the Hospital for Children last year, the opportunity presented itself for observing several cases, in which the aveolar processes had so disappeared that in children of from four to six years of age, after the primary teeth had fallen out, the germs of the developing permanent teeth were exposed to view.

In the upper jaw, the destroying process does not proceed to such a depth as in the lower jaw, because the ichorous matter, being heavy, has a tendency to sink downward, and thus considerably facilitates the cleansing of the parts; while, in the lower jaw, the decomposing fluid is retained in the alveoli and in the porous bones, thereby greatly favoring the extension of the disease. Frequently, necrosed portions of the alveolar process, an inch in length, are exfoliated. Necrosis does not always proceed from the alveolar border downward; but the gums may be destroyed and the anterior bony wall die away, leaving the dental germs exposed.

This disease occurs more freqeently in children than in adults, and more often in those who have lately recovered from tedious illness—as scarlet fever, measles, and typhus—than in those who are otherwise quite healthy.

A chilly, damp dwelling is commonly an exciting cause, or exposure to a cold, damp draught: the latter is often the sole cause of such disease in healthy and often strong men, as travelers, or those engaged in business in wet localities. The disease only assumes an epidemic character in hospitals for children. This appears to arise partly from immediate contagion, through the transmission of the exudation from one child to another by means of various articles in use, and partly also from the weakness which follows long-continued illness. Sometimes a peculiar tendency does not appear to lie in any definite external circumstances; for it is a fact that, in the sick-chambers set apart for these patients, their number will not diminish for several weeks together, but recruits are constantly added from other wards, while the earlier patients have long since been dismissed as cured.

The duration of this disease is dependent on its extent, especially on the stage in which treatment is first resorted to, and, further, on the general state of the patient's health Its effects are generally more lasting and destructive when it succeeds a previous illness than when occurring in healthy individuals. Slight cases are curable in from eight to fourteen days, the more severe requiring often several months.

*Treatment.*—As croupy inflammation of the gums exerts its hurtful effects particularly through the lengthened presence of the ichorous product discharged from its generating source on the surrounding parts, the first thing suggesting itself as favorable to a cure is extremely careful cleansing. The best plan is carefully to remove the exudation before it is thrown off. This is readily effected, and that without giving much pain, by removing the exudation, especially from between the teeth, with a dossil of lint held with an instrument. This done, the ulcerated surface is cleansed from blood by syringing with warm water: a little alum may be added when the hæmorrhage is excessive. When the mucous membrane is thus cleansed, the wounded surface should be touched with caustic, not forgetting to apply it well between the teeth. In the interim, the patient should frequently take warm mucilaginous decoctions in the mouth, which should be a cleansing and soothing kind; and the cauterizing must be repeated so long as any new exudation is formed.

As this disease calls for direct treatment, which is very often refused, especially by children, because it is accompanied with pain, and the nurse, and often even the doctor, avoids the patient, on accounl of the dreadful fetor, very neglected cases of this kind are constantly received for treatment. An ill-smelling fluid, a mixture of saliva and ichor, flows from the mouth; the lips and inferior maxillary glands are swollen; the otherwise indolent patient announces his approach to the doctor in tears and fear of coming pain. In such cases, the mouth should be well syringed with a slightly-aromatized warm fluid, in order to remove all loose matter. The patient, who feels an immediate benefit from this cleansing, soon endeavors to open his mouth, when all loose flakes of exudation can be removed, and the state of the teeth ascertained. If the teeth are found to be very loose, and ichorous matter flowing from the alveoli, the teeth must be removed, especially if they are carious, or, as is often the case in children, the roots are exposed. If any necrosed portions of bone are discovered, they should be removed at once. After checking the hæmorrhage, the ulcerated portions should be cauterized.

Besides this local treatment, the 'general' is confined to change of air, and, in the convalescent, a liberal diet. By careful attention to this method of treatment, no trouble being spared, the disease can be cured, and its effects limited; though a great extension of the disease in weak persons, particularly in neglected children, may terminate in death through absolute exhaustion."—*London Review—Dental Cosmos.*

